# Edge-illumination x-ray phase-contrast imaging

**DOI:** 10.1088/1361-648X/ac0e6e

**Published:** 2021-07-13

**Authors:** Alessandro Olivo

**Affiliations:** 1 Department of Medical Physics and Biomedical Engineering, UCL, London, United Kingdom

**Keywords:** x-ray imaging, phase-contrast, x-ray masks, structured illumination

## Abstract

Although early demonstration dates back to the mid-sixties, x-ray phase-contrast imaging (XPCI) became hugely popular in the mid-90s, thanks to the advent of 3rd generation synchrotron facilities. Its ability to reveal object features that had so far been considered invisible to x-rays immediately suggested great potential for applications across the life and the physical sciences, and an increasing number of groups worldwide started experimenting with it. At that time, it looked like a synchrotron facility was strictly necessary to perform XPCI with some degree of efficiency—the only alternative being micro-focal sources, the limited flux of which imposed excessively long exposure times. However, new approaches emerged in the mid-00s that overcame this limitation, and allowed XPCI implementations with conventional, non-micro-focal x-ray sources. One of these approaches showing particular promise for ‘real-world’ applications is edge-illumination XPCI: this article describes the key steps in its evolution in the context of contemporary developments in XPCI research, and presents its current state-of-the-art, especially in terms of transition towards practical applications.

## 1. Origins of x-ray phase contrast imaging

Fritz Zernike’s development of the phase-contrast microscope changed the history of science, and earned him the Nobel Prize [1]. Translating this momentous achievement to the x-ray regime was no simple feat, primarily because of the significantly increased difficulties in manipulating x-ray beams with optical elements, compared to visible light beams. In 1965, Bonse and Hart proved this was indeed possible [2], by developing an interferometer based on three perfect crystals. A significant simplification came through the use of a single, post-sample ‘analyser’ crystal (although the beam typically needs to be ‘prepared’ by at least one additional crystal upstream of the sample). This was indeed at the basis of one of the pioneering papers which contributed to the ‘explosion’ of x-ray phase contrast imaging (XPCI) in the mid-90s [3], although it had been proposed already in the ‘80s in (up to that point) relatively little known papers [4, 5]. An additional, significant simplification came in the shape of a propagation-based (PB) approach, effectively corresponding to a form of ‘in-line holography’ for x-rays, which required no optical elements, but simply the propagation of a partially coherent wave over some distance downstream of the imaged object [6, 7]. The need for a degree of spatial coherence, i.e. for a small focal spot source placed at some distance from the sample, is the main limitation of the PB approach, which was indeed demonstrated either with synchrotron [6] or micro-focal x-ray sources, the latter leading to exposure times of the order of hours also for relatively small objects [7]. Furthermore, detectors with a relatively small pixel size are typically required to resolve the fringes created by the interference between the differently phase-shifted wavefronts that travelled through a certain detail and immediately outside it [8]. However, due to its inherent simplicity, PB XPCI is still one of the most widely used methods in research; an additional advantage comes from its allowing the use of polychromatic spectra [7], with these having only a limited effect on image contrast compared to the much stronger one caused by the finite focal spot size [9]. When implemented with focal spot and detector pixel sizes comparatible with e.g. clinical practice, however, the advantages in image contrast brought by phase effects tend to vanish [10].

Converesly, methods based on crystal analysers allow for the use of larger focal spots [11], but crystals monochromatise the beam, which means that only a small fraction of the spectrum produced by a conventional x-ray source is used for imaging, again leading to excessively long exposure times. For this reason, alongside the relatively complex experimental setup, the use of crystal-based methods has somewhat declined over recent years. However, their use allowed some of the milestones in the development of XPCI. These include some of the earliest examples of quantitative separation of phase and attenuation (‘phase-retrieval’) in the ‘90s [12], and the first quantitative extraction of the dark-field (or ultra-small angle x-ray scattering, USAXS) contrast channel in the early 00s [13–15]. Performing quantitatively exact phase retrieval with PB methods is more complex, and requires the acquisition of multiple images at varying sample-to-detector distances [16]. Simplified ‘single-image’ approaches exist [17] that yield quantitatively correct results on homogeneous objects, and good image quality (albeit at the cost of quantitativeness) on samples with limited inhomogeneity.

### 1.1. Advantages of x-ray phase contrast imaging

XPCI is based on a different physical phenomenon compared to conventional x-ray imaging—phase changes rather than attenuation differences. A simple way to describe this is by referring to the complex refractive index:

\begin{equation*}n=1-\delta +\mathrm{i}\beta \end{equation*}

where *β* drives the attenuation effects, and *δ* is responsible for the phase shifts. In the energy range of x-rays, and especially for biological materials, *δ* is much larger than *β*, typically by 3 orders of magnitude (e.g. for a plastic such as C_2_H_4_, values at 25 keV are 3.5 × 10^−7^ and 8.1 × 10^−11^, respectively). It is important to note that this does *not* mean that the contrast is increased by 3 orders of magnitude, as the real contrast increase depends on the sensitivity of the used XPCI method and on the specific sample. However, significantly higher contrast between different materials is typically observed, with contrast increases of up to 2 orders of magnitude being within reach, especially at synchrotrons [18–20]. Such an increase in contrast leads to a proportional increase in signal-to-noise ratio (SNR) for the same detected x-ray statistics. This means that details presenting negligible attenuation differences against the background they are immersed in, and that would therefore be considered ‘invisible’ to conventional x-rays, can be detected by XPCI, which effectively extends the application remit of x-ray imaging, and opens the way to its use in areas which were previously considered inaccessible.

One of the most relevant examples is the discrimination between different biological soft tissues, which was identified as a target already in the early days of XPCI development [21]. Among these, the detection of breast tumours [8, 22, 23], lung diseases [24–26] and cartilage degeneration [27, 28] rapidly assumed particular prominence, and indeed all these fields are currently moving into *in vivo* experimentation, either with synchrotron radiation [29] or conventional sources [30, 31]. Applications were rapidly identified outside biomedicine e.g. in materials science [32, 33] and cultural heritage [34]; the application list is so long it would require an article on its own merit, and readers are referred to some recent reviews [35–38] for more details.

The ‘increased contrast leading to an increased SNR for the same detected x-ray statistics’ (i.e. for the same dose) argument can be turned on its head—achieve the same SNR with a lower statistics—and indeed indications that XPCI can be used to significantly reduce the dose delivered in x-ray examinations have been provided [39]. *δ* also decreases more slowly than *β* with increasing x-ray energy, which means that e.g. soft tissue contrast can be maintained at more penetrating, higher x-ray energies that deposit a reduced amount of dose in the tissues themselves [40].

### 1.2. X-ray phase contrast imaging with laboratory x-ray sources

As already mentioned implementing XPCI with laboratory sources is relatively straightforward, and indeed some of the pioneering papers were based on research performed with x-ray tubes [2, 3, 7]. The key issue is the flux limitation leading to exposure times too long to be practical, caused either by monochromatisation by a crystal [2, 3] or spatial coherence requirements imposing the use of a microfocal source [7].

Indeed when a new class of laboratory-based methods was introduced in the mid-00s, the emphasis was put on the use of ‘low brilliance’ x-ray sources [41]. Two methods were developed in rapid succession—‘grating’ based XPCI [41] and edge-illumination (EI) [42], the subject of this paper.

Grating or ‘Talbot’ interferometry was enabled by advances in microfabrication methods allowing the development of gratings with a small enough pitch to produce Talbot ‘self-imaging’ effects [43] at x-ray wavelengths. Phase changes induced by a sample perturbe the self-image, and appropriate recording of these perturbations enables phase retrieval. Also in this case, this was initially explored with synchrotron radiation: following pioneering investigations by Cloetens *et al* [44], proof-of-concept results were provided by David *et al* [45] and by Momose *et al* [46]. The transition to low-brilliance sources is operated by switching from a ‘Talbot’ to a ‘Talbot-Lau’ configuration. The latter consists of the introduction of an additional (absorption) grating in front of an extended x-ray source, so as to slice it into an array of mutually incoherent, but individually coherent x-ray ‘sourcelets’ [41]. The ability of this method to operate in CT mode [47] and to provide access to the USAXS channel [48] like its synchrotron predecessors were rapidly proven, and a significant enhancement in sensitivity was obtained by switching from micro to nanogratings [49].

EI on the other hand originates from earlier research on ‘edge-on’ silicon microstrip detectors performed at the Elettra Light Source in Trieste, Italy [50], and from the subsequent observation that hitting detectors pixels only on their edge with a collimated beam significantly enhances their sensitivity to phase effects [18].

## 2. Edge-illumination x-ray phase contrast imaging

**Figure 1.**
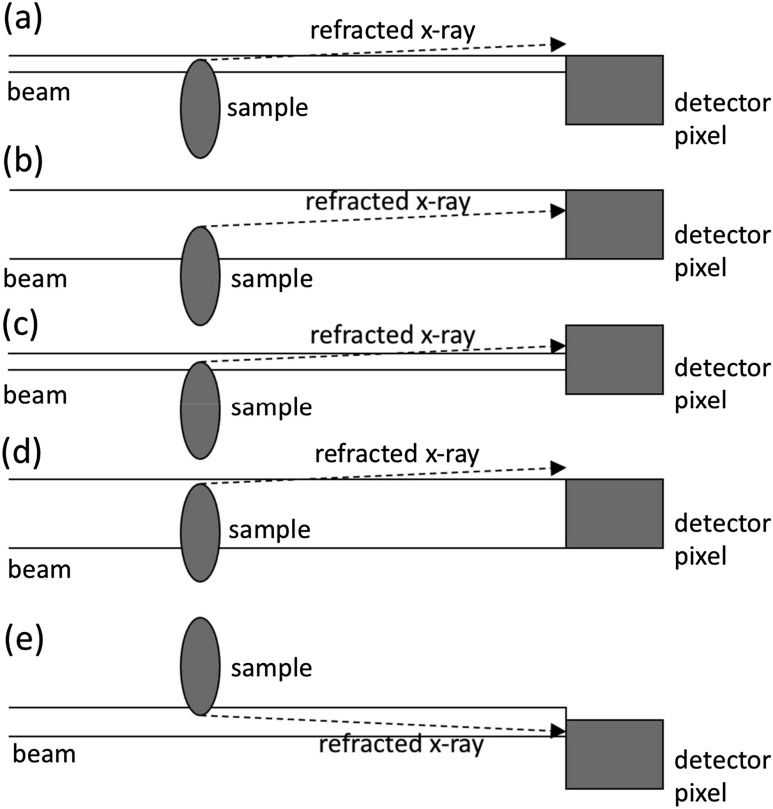
Edge-illumination XPCI setups creating single negative fringes (a) and pairs of positive/negative fringes (e) along the border(s) of object details, compared with cases where these are not created, either with thick (b) or thin (c) beams. (d) Represents a situation that does create a signal, but in which this tends to be washed out by a much larger background (see text for details).

A simplified but effective [51] way to describe phase effects is by using ray-tracing, and referring to x-ray refraction. To first approximation, the refraction angle *α* is proportional to the first derivative of the phase shift:

\begin{equation*}\alpha \cong \frac{\lambda }{2\pi }\vert { \overrightarrow {\nabla }}_{x,y}\phi \vert \end{equation*}

where *λ* is the x-ray wavelength, *x*, *y* define the plane orthogonal to the (original) x-ray propagation direction *z*, and *ϕ* is the phase shift introduced by the object, defined as:

\begin{equation*}\phi =\frac{2\pi }{\lambda }{\int }_{\text{object}}\delta \left(x,y,z\right)\mathrm{d}z\end{equation*}

where the integral extends over the entire thickness of the object along the x-ray propagation direction *z*. This means that making an x-ray imaging system sensitive to phase effects corresponds to making it sensitive to refraction, which is indeed what original crystal-based methods do, thanks to the extremely high angular selectivity of perfect crystals to monochromatic x-rays [3, 12]. The original synchrotron EI implementation achieves this by collimating the beam in the vertical direction, and aligning it with the edge of a detector pixel (figure 1). As can be seem in figure 1(a), the limited thickness and the proximity to the pixel edge makes it very easy for a refracted photon to ‘escape’ detection by a given pixel, leading to a detectable reduction in the number of counts. Figure 1(b) shows that the same refraction angle does not lead to the same effect if the beam is aligned with the centre of the pixel, even if its vertical thickness is made much smaller than the pixel itself (figure 1(c)). Clearly the effect still exists with a ‘larger’ beam when a photon that would hit the pixel close to its physical edge is considered (figure 1(d)), however this leads to a much smaller relative signal, since most of the beam would not be deviated outside the pixel: the signal created by the few photons at the periphery of the beam would be ‘washed out’ by the vast majority of the ‘central’ ones, which would still be detected even if deviated by the sample. Finally, making the beam slightly thicker so as to reach outside the pixel allows for the creation of positive signals when the opposite edge of the sample is hit (figure 1(e)), which effectively ‘doubles’ the detected signal by creating a positive fringe on one side of the sample as well as a negative one on the other.

While the above discussion refers generically to a ‘sample’, the same concept applies to refraction generated at the interfaces of all details inside a larger object, i.e. the ‘sample’ represented in figure 1 can be equally imagined as a small feature embedded in a much larger background.

As a synchrotron embodiment, this is a ‘scanning’ method. The ‘pixel’ represented in figure 1 can be imagined as a 1D detector (effectively a row of pixels) entering the plane of the drawing; the object is then scanned upwards or downwards, and an image line acquired for every object position. Alongside sensitivity to very small refraction angles (∼2 nanoradians, [20]), this ‘scanning’ approach provides easy access to ‘single-scan’ USAXS imaging by shifting the entire thin beam immediately outside of the pixel [52], albeit at the cost of some contamination with the other contrast channels. Through use of a multi-layer detector thin enough to be fully illuminated by the synchrotron beam in the vertical direction, beam-splitting through a mask allowed arguably the earliest simultaneous ‘multi-modal’ acquisitions [53].

**Figure 2.**
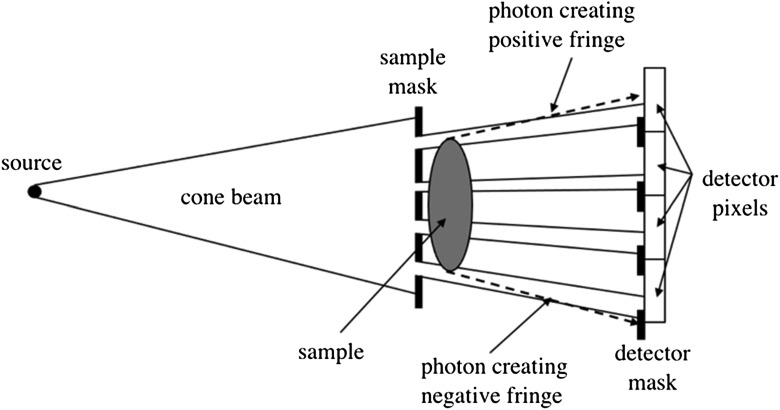
Adaptation of EI XPCI to cone beams produced by laboratory sources and area detectors by means of a pair of opportunely designed masks, essentially identical apart from a scaling factor that accounts for the beam divergence. Note the two masks are slightly displaced with respect to each other, so as to reproduce the EI condition shown in figure 1(e) for every row (or equivalently column) of the 2D detector.

Indeed, beam-splitting through masks inspired the adaptation of the technique to conventional x-ray sources, by individually ‘edge-illuminating’ every line of a 2D (area) detector with a structured cone beam (figure 2).

A second mask was used in these initial experiments to create insensitive regions between adjacent pixels; options to use a single mask are discussed in section 3. However, the use of two masks has the significant advantage of enabling the use of practically any detector, regardless of its pixel size and width of the pixel’s point spread function (PSF); for this reason, we still use it frequently with e.g. indirect conversion flat panels. The pixel size only affects image resolution and not phase sensitivity, since this is determined at the level of the individual beamlet regardless of how spaced apart these are: so long as the bemlets are kept sufficiently separated from each other, the mask period has no effect on the phase sensitivity [54], a significant difference from e.g. grating interferometry, in which the sensitivity depends directly on the grating period [55].

Resolution can be improved through a ‘dithering’ approach, in which multiple frames are collected while the sample is displaced in sub-pixel steps, and recombined to create an image oversampled at such a ‘dithering’ step. While normally this approach would require some degree of post-processing of the recombined image (e.g. deconvolution [56]), in EI this is made unnecessary by the masks redefining the resolution properties of the detector. Indeed, with an appropriate dithering step, the resolution in the final image is equal to the size of the apertures in the sample mask [57].

A PSF with a full width at half maximum significantly larger than the pixel can, on the other hand, generate artefacts when combined with dithering [58]. To mitigate this, ‘skipped’ masks, illuminating every other detector row (or column), are normally employed with detectors with very broad PSFs. This effectively provides a means to implement EI with any detector; however, if the PSF extends over several pixels, then skipping too many pixel rows or columns would lead to a more inefficient use of the beam, and/or to the need to use an increased number of dithering steps to recover the desired resolution level.

Finally it should be noted that, for simplicity’s sake, apertures corresponding to (vertical or horizontal) long slits have been discussed so far. These result in phase sensitivity along one direction only, namely orthogonally to the slits themselves. However solutions where each pixel is simultaneously ‘edge-illuminated’ along two directions are easily devised, for example through arrays of L-shaped apertures matching each pixel [59].

### 2.1. Retrieval methods in edge illumination XPCI

A single-shot image captured with a system like that schematised in figure 2 contains a mixture of attenuation and phase, with phase-enhancing features superimposed to the conventional attenuation image. For applications which simply require enhancing the visibility of specific details, this may be sufficient [8, 60–62], and this is indeed the approach followed by the *in vivo* mammography study with synchrotron radiation underway at the Elettra Light Source [29].

It is however possible to separately retrieve phase and attenuation images, i.e. separate maps of (‘effective’ when polychromatic sources are used [63]) *δ* and *β*.

Since two unknowns need to be retrieved, this typically requires two input images. The first retrieval methods were inspired by Chapman *et al*’s pioneering work with crystals [12], and based on acquiring two images while the beamlets illuminate opposite sides of the apertures in the detector mask (imagine acquiring a first image in the configuration shown in figure 2, and a second one after moving the sample mask upwards by a step equal to the size of its aperture). This ‘switches’ the phase signals: in the first case, photon deviated upwards (downwards) increase (decrease) the number of counts, while in the second case (i.e. after the mask is displaced) the opposite occurs; attenuation obviously remains unaffected. An analytic formula can then be used to separate the two [64]; note that, since EI is sensitive to refraction, the first derivative of *δ* in the direction orthogonal to the slits will be retrieved, and this can then be integrated if a map of *δ* itself is required [65].

**Figure 3.**
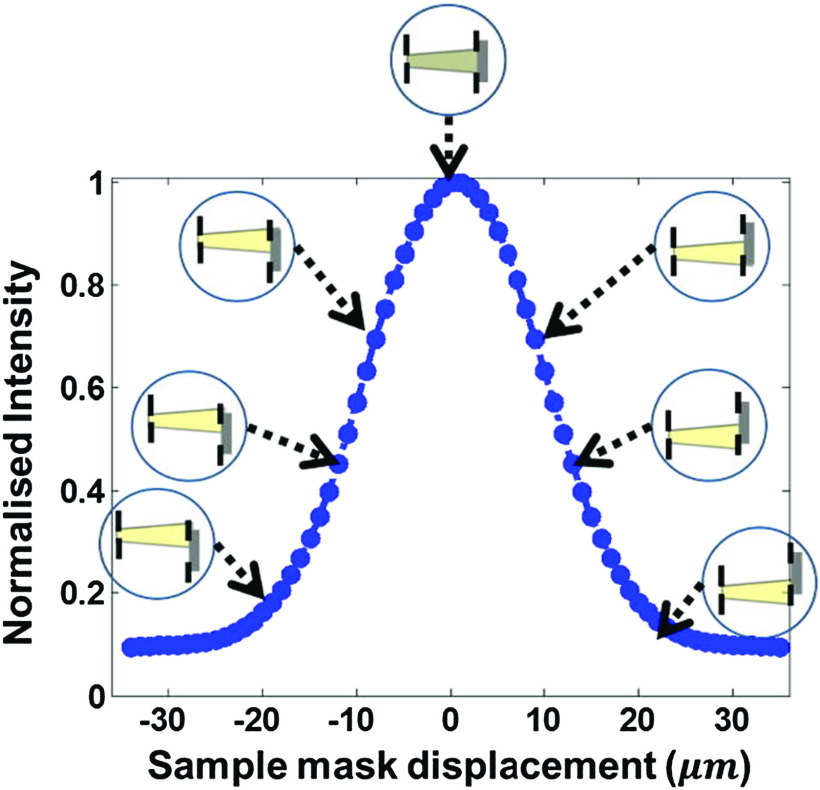
The ‘illumination curve’ obtained by scanning one mask while the other is kept still represents the system’s sensitivity and can be used to perform phase retrieval.

A simplified approach to phase retrieval, however, comes from mathematically inverting the ‘illumination curve’ (IC), i.e. the curve that is obtained by scanning the sample mask while keeping the detector mask stationary, and recording the x-ray intensity detected at every position (figure 3). When a beamlet is refracted by a sample, the detector/detector mask combination will ‘perceive’ it as a commensurate displacement of the sample mask, i.e. a displacement of the illumination point on the IC. The IC therefore ‘maps’ refraction in terms of variation in detected intensity, and therefore its mathematical inversion (after conversion of the horizontal axis in figure 3 from linear displacement to angles) can be used to retrieve refraction from intensity changes [66]. Obviously single images are still mixed, therefore a minimum of two frames is still required to separate out the attenuation contribution. Phase retrieval can equally be implemented in 2D, provided an appropriate mask design is used [67].

Phase retrieval is an essential step to perform computed tomography [68]. One downside of combining phase retrieval and CT is the need for multiple input frames at each projection angle, which prevents a continuous rotation of the sample and imposes a ‘step and shoot’ approach, which inevitably lengthens the acquisition time. 3D ‘mixed’ images, where phase effects enhance attenuation features, can be directly reconstructed if the rotation axis is orthogonal to the mask apertures’ direction [69]; however, the separate availability of *δ* and *β* can be extremely useful in e.g. material identification [70]. One option to retrieve both quantities with a continuous rotation of the sample is offered by the ‘reverse projections’ approach, based on the observation that a projection acquired at angle *α* + 180° correspond to that acquired at *α* with the beamlets created by the sample mask illuminating the opposite side of the apertures in the detector mask [71]. The idea was adapted from previous synchrotron experiments carried out with analyser crystals first [72], then gratings [73]. Therefore, (continuous) acquisitions are performed over 360° instead of 180°, and retrieval is performed on pairs of projections separated by 180°, before CT reconstruction over 180°. However, the underpinning assumption is strictly valid only with a parallel beam, hence artifacts appear if a cone beam is used; moreover, the axis of rotation must be pre-aligned with the centre of a pixel. This can be solved through appropriate iterative reconstruction approaches [74, 75], which tend to be more computationally expensive; however, they also offer the option to reduce the number of acquired projections [76] and/or dithering steps if an increased in-slice resolution is required [77, 78].

Moreover, Paganin’s pioneering single-shot approach [17] can be adapted to EI [79], which allowed the fastest CT acquisitions with a laboratory source on record [80], opening the way to some of the most important applications discussed in the next chapter.

**Figure 4.**
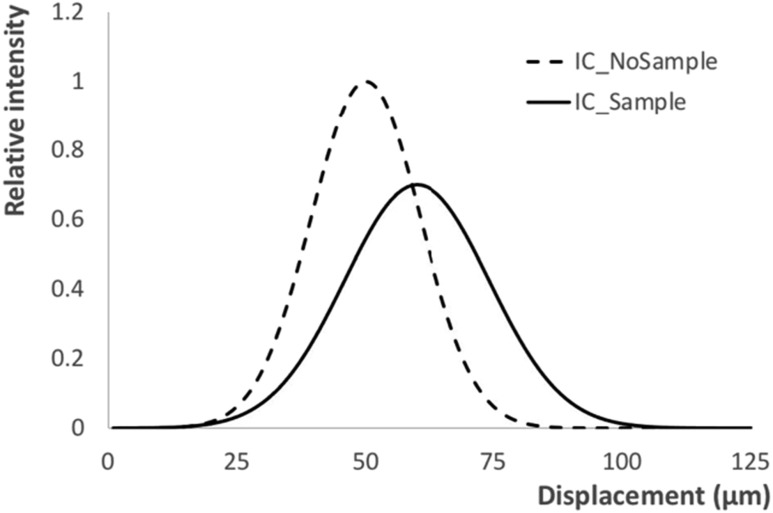
Illumination curve before (dashed line) and after (solid line) the introduction of a sample. In general terms, this causes a dampening, a lateral shift and a broadening of the IC, corresponding to attenuation, refraction (i.e. phase) and dark field (or USAXS), respectively.

So far, refraction and attenuation have been considered, which correspond to a lateral displacement and a dampening of the IC. A third phenomenon can be observed in the presence of a sample, namely a broadening of the IC (figure 4).

This broadening appears if the sample is inhomogeneous on a scale below the spatial resolution of the imaging system, in perfect analogy with the broadening of the rocking curve first observed with crystal-based methods [13–15], and later translated to grating interferometry in terms of reduction of the fringe visibility [48]. Its extraction alongside attenuation and refraction requires the collection of a third frame [81], typically acquired with the apertures in the two masks mutually aligned (i.e. on the ‘top’ of the IC), as this optimises dose efficiency. The retrieved broadening can be quantitatively correlated to the degree of (sub-resolution) inhomogeneity that caused it through a relation that can be cast as a line integral, which allows performing dark field CT [82].

One downside is the need to acquire three separate images; however, at least in scanning-based approaches where the sample is laterally translated through the beam (imagine scanning the sample figure 2 upwards or downwards), this could be avoided through the adoption of an ‘asymmetric’ sample mask [83]. In an asymmetric design, groups of apertures are shifted from their original position, so that the corresponding beamlets hit the apertures in the detector mask at different positions. The simplest design is a ‘three-way’ asymmetric mask, where:

• Apertures 2, 5, 8 etc are left in their original position: when the mask is aligned, the corresponding beamlets will hit the centre of the apertures in the detector mask (‘top of the IC’);
• Apertures 1, 4, 7 etc are pulled backwards so that corresponding beamlets hit the top edge of the detector mask apertures (50% of the IC on the left-hand side);
• Apertures 3, 6, 9 etc are pushed forward so that the corresponding beamlets hit the bottom edge (50% of the IC on the right-hand side).

Following a scanned acquisition, detector columns corresponding to the above groups are combined to form the three separate images which are required by the retrieval algorithm. Effectively, detector area is traded off for images acquired at different positions of the IC, with ‘hypothetical’ detectors with a third of the area consisting of columns 1, 4, 7, …; 2, 5, 8, … and 3, 6, 9, … respectively providing the images required by the phase retrieval process. Since they originate from different detectors columns, these images are shifted with respect to each other, and need to be re-aligned before being given as input to the phase retrieval algorithm. This approach makes it very easy to reach large fields of view, by developing masks which are narrow in the direction of the scanning, and long in the other [84]. Sample-free areas in the scans can be used as a reference to make sure the mask position has not shifted with respect to the ‘targeted’ IC value, or to correct the retrieval accordingly.

The three-image retrieval method ‘condenses’ the USAXS signal in a single parameter, namely the amount of broadening of the IC; however, it has been proven that changes in the shape of the IC can also yield useful information in various applications [85–87], extracting which requires the acquisition of more than three frames.

### 2.2. Applications of edge illumination XPCI

Early applications of EI XPCI were based on single-shot, 2D ‘mixed’ images, and aimed at areas of medical imaging where conventional x-rays have limitations. One obvious target is cartilage, which is almost transparent to conventional x-rays and therefore very difficult to visualise. Indeed, the distance between phalanges is used as a proxy for cartilage thickness in basic studies, while MRI has to be used in more complex cases.

**Figure 5.**
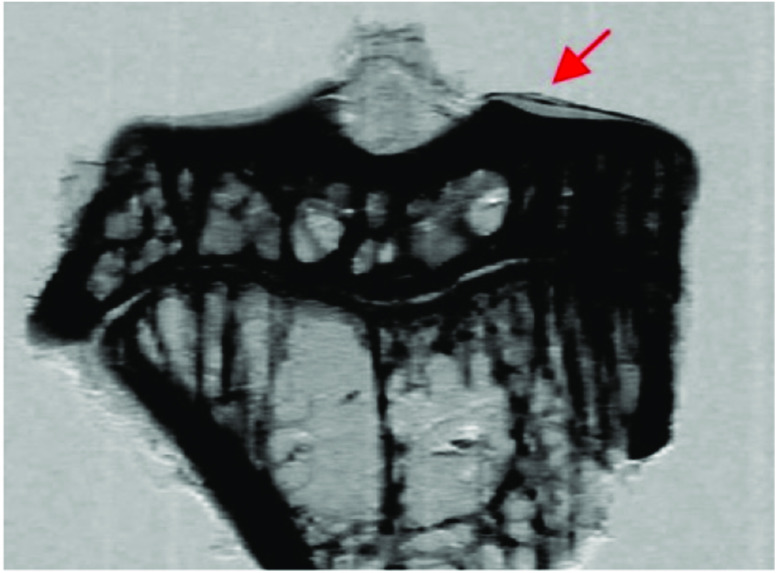
An example of (mixed, single-shot) EI image of a slice through a mouse knee, in which the cartilage layer is made clearly visible by the (dark) refraction fringe at its edge. This makes it easier to detect small damage, as its presence causes a small discontinuity in the refraction fringe. In this example, the arrow points at a minor interruption in the fringe, which was confirmed to be a small lesion through histology [61].

Our preliminary study on murine cartilage showed that EI XPCI can clearly visualise the hyaline cartilage layer, as well as detect minor lesions therein [61]. A successive study showed compatible detectability of cartilage layers to the synchrotron gold standard [88], which had been used successfully before for the same purpose [27, 28]. The ability to provide cartilage visualisation comparable to synchrotrons with a conventional x-ray source can have applications in pre-clinical and, in the longer term, clinical imaging. Pre-clinical imaging is important because most drug development work aimed at treating osteoarthritis, for which currently there is no cure except prosthetic surgery, is based on small animals, whose cartilage is invisible in conventional small-animal CT scanners. Scaling up the technology could then allow detecting early cartilage damage in human patients, increasing the chances of a cure or at least palliation leading to better life quality. An example of visualisation of cartilage damage in a mouse knee with laboratory-based EI XPCI is shown in figure 5.

Another key area of application is mammography, which is a challenging field for conventional x-ray imaging because of the low soft tissue sensitivity of conventional x-rays, and in particular of the small attenuation difference between healthy and diseases breast tissue [23].The ability of XPCI to allow the detection of previously undetectable lesions has already been demonstrated through synchrotron studies [8, 22, 23]. Once XPCI with conventional sources became feasible, the possibility to achieve the above advantages with a device that could be deployed in the clinics started to be investigated. As well as showing enhanced detection of several tumour-related features such as calcifications, EI XPCI was the first lab-based method to show that this could be done while delivering to the organ radiation dose levels compatible with clinical practice [62], which had proven challenging in previous attempts [89].

**Figure 6.**
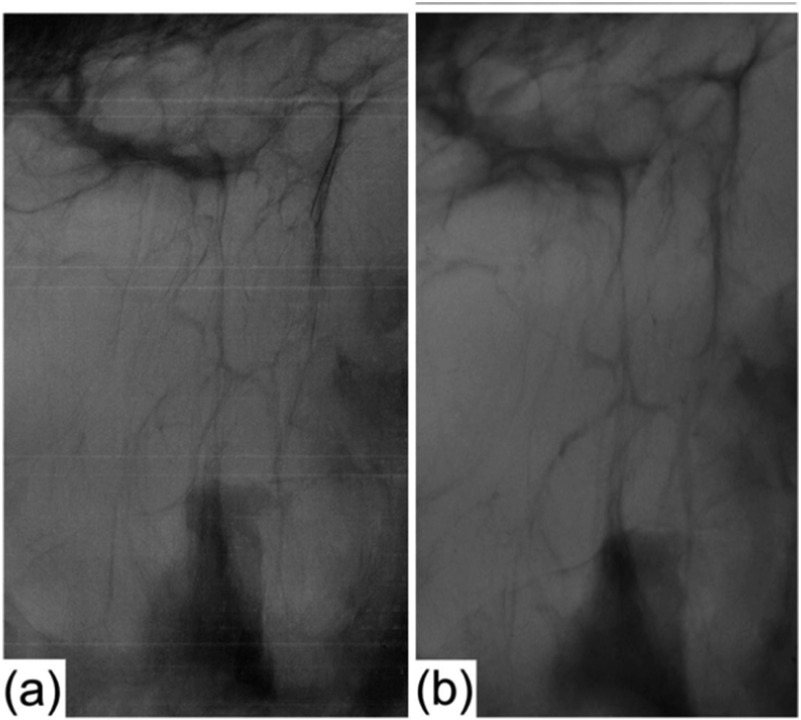
Stomal trabeculae in breast tissue (along which tumours tends to spread) visualised by EI XPCI (a) and conventional x-rays (b).

Subsequently, potential for significant dose reduction was demonstrated in a synchrotron experiment [40], initial translation of which to a lab source was later demonstrated through a phantom study [84]. Figure 6 shows an example EI XPCI image of breast tissue compared to a conventional x-ray attenuation one. This example focuses on the enhanced detection of stromal trabeculae and thin tissue strands in general. As well as in diagnostic imaging (e.g. to determine the extent of the tumour), this has great importance in intra-operative imaging applications, where the surgeon needs to determine, ideally in real time, whether the entire tumour has been resected. The adaptation of EI XPCI to this application is discussed below.

Still in terms of 2D (planar) imaging, the retrieval of the shape as well as the width of the IC showed potential to discriminate between healthy and emphysematous mouse lungs [85]; once again, lung imaging is an area where pilot studies had already indicated significant potential [24–26]. Finally, the use of microbubbles as a novel x-ray contrast agent is another area previously explored by our and other groups [90–92], including dynamically [93]. A pilot experiment at a synchrotron recently demonstrated that EI allows quantifying microbubble concentrations also at high x-ray energy [94], a result we are now planning to translate to standard labs.

**Figure 7.**
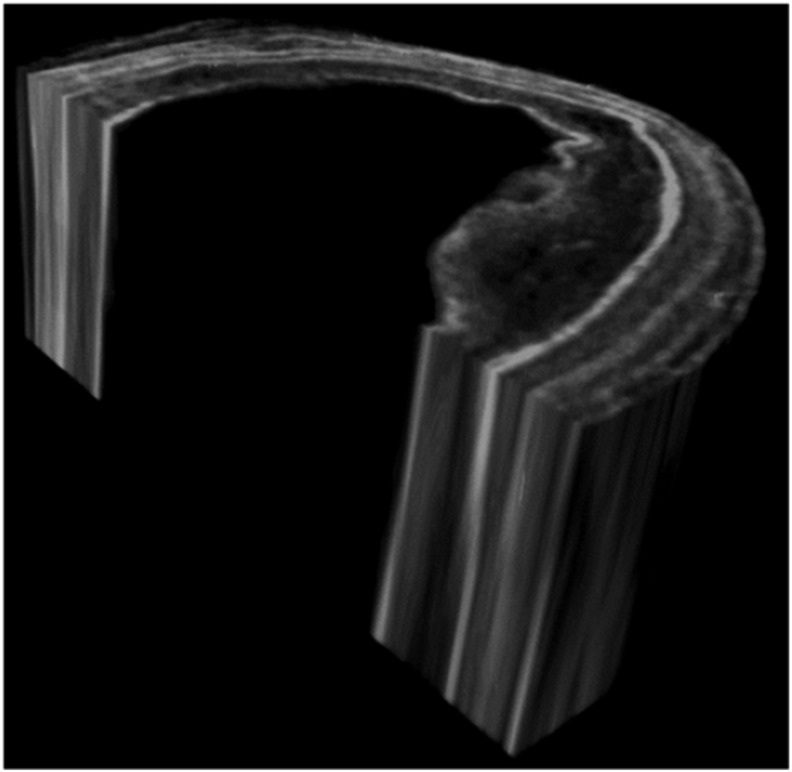
An example of EI CT’s ability to resolve the various tissue layers (clearly visible in the cross-section) in a scaffold obtained by decellularising a piglet’s oesophagus. This enables assessing the integrity of each layer, a fundamental pre-requisite for the ensuing re-cellularisation process.

The adaptation of EI XPCI to CT opened the way to a significant number of new applications, notably tissue engineering, where it offers a non-destructive mechanism to assess the integrity and viability of e.g. scaffolds based on native tissue following processes of de- and re-cellularisation [95]. Imaging is of paramount importance to drive the development of regenerated organs, and indeed it is used at various stages of scaffold creation and to study its interaction with the implanted cells. At the moment, histology is used as the gold standard, however it is destructive, and extracting any degree of three-dimensional information is both laborious and challenging. Magnetic resonance imaging and x-ray micro-CT offer possible alternatives, however the former suffers from limits in resolution, long acquisition times and high costs, while the latter lacks the necessary soft tissue contrast. XPCI CT could fill this gap by providing a ‘best of both worlds’ approach where the soft tissue sensitivity of MRI is combined with the high resolution, speed and cost effectiveness of x-rays. Not only did the cited study demonstrate that this is indeed possible, but it also showed compatibility between lab images and the synchrotron gold standard based on single-shot Paganin retrieval [17]. An example of the ability of EI XPCI to resolve the various tissue layers in a scaffold obtained by de-cellularising a piglet’s oesophagus is shown in figure 7.

A boost to the range of applications accessible to phase-based CT came with the extension of Paganin’s algorithm [17] to the EI approach, especially the translation from synchrotron [79] to laboratory sources [80]. The ‘single-shot’ capability enables performing phase retrieval on each CT projection, while keeping the masks in a fixed position, and continuously rotating the sample (‘flyscan’). This eliminates all dead times and overheads (apart from the detector readout time, negligible in many of the currently available devices), ultimately allowing to perform a phase CT with a lab system in a matter of a few minutes. This opens the way to applications where fast scans are of the essence, such as for example intra-operative and pre-clinical imaging.

A reliable intra-operative imaging solution is particularly needed in breast conserving surgery, where resected tissue (the ‘wide local excision’, WLE) is sent to the pathology lab for assessment, with results becoming available several days after the operation. When histopathological analysis reveals cancer presence at the margins of the WLE, this can result in a re-operation, which significantly adds to the patients’ anxiety, can affect their wellbeing, and increases the costs incurred by health services. XPCI’s ability to provide an enhanced visualisation of cancer lesions in breast tissue [8, 22, 23, 29, 62] was combined with EI’s ability to perform fast phase-based CT to develop a solution to this problem. Following demonstration that building a compact system that would fit the requirements of operating theatre installation while maintaining the required levels of sensitivity and resolution was indeed possible [96], we ran some preliminary tests on tumour-bearing breast tissue specimens [97], the success of which triggered a larger statistical study, the results of which are currently under review. Concurrently, we are exploring the advantages of our intra-operative imaging approach in other fields, e.g. oesophageal surgery.

While many intra-operative applications are a perfect match with Paganin-type single-shot retrieval because the specimens are, to a good approximation, homogeneous in composition, pre-clinical (or ‘small animal’) imaging poses an additional challenge, because the simultaneous presence of bone and soft tissue breaks Paganin’s ‘homogeneity’ assumption. To tackle this, we developed an adaptation to EI [98] of Beltram *et al*’s approach [99], which deals with this problem by reconstructing interfaces one at a time by using a different *δ* to *β* ratio for each, then splicing together the results. Preliminary studies on small animal imaging have been conducted with promising results [100, 101], which led to additional research currently underway.

**Figure 8.**
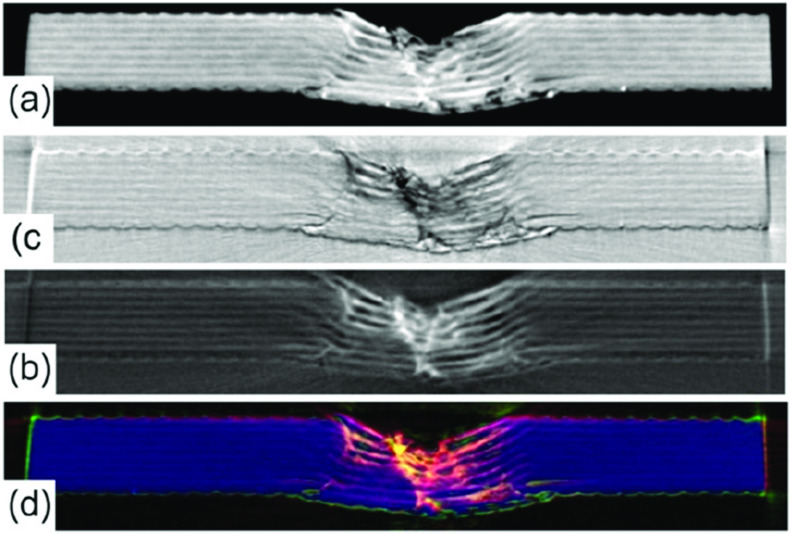
Complementarity of the various contrast channels provided by EI demonstrated on a CT slice taken through the damaged part of a composite material sample. Panels (a)–(c) show attenuation, refraction and dark-field CT slices, respectively. Panel (d) combines them in a single RGB image (with red, green and blue used for dark-field, refraction and attenuation, respectively), showing how they all emerge from different parts of the sample, especially as far as the damaged region is concerned.

More recently, the list of applications of EI has expanded to include several non-medical areas. One of the first non-medical areas to be targeted was materials, especially composites—initially by showing dark-field’s ability to reveal damage undetected through other means [102], and more recently by analysing the complementarity between the different contrast channels (see figure 8), and showing how these extend the results achievable with conventional micro-CT [103]. Carbon fibre reinforced composites are widely used in the aerospace industry due to their high strength-to-volume ratio. However, they are prone to barely visible impact damage, which can be difficult to detect with established techniques. The ability to detect defects and early signs of damage is important both when new approaches to the fabrication of composites are developed, and especially to test existing components to prevent their possible failure during operation. Albeit still preliminary, the results obtained in the above studies indicate that early matrix cracks can be detected by dark-field, and that the complementarity between contrast channels can help identifying and separating different types of damage.

The ability of the dark-field channel to detect individual sub-pixel features (alongside ‘averaged’ signals from inhomogeneous sample regions) was also recently demonstrated, and it was shown how this could be used to detect micro-damage in electronic components [104].

Other non-medical applications are made possible by the technique’s resilience against high x-ray energies, which was highlighted already in the early days of its translation for use with conventional sources [105, 106], and confirmed through some pilot synchrotron studies [19, 20]. These include security, early results on which [60] triggered a larger study on threat materials currently underway and, to a minor extent, paleontology [19]. More recently, EI XPCI was also included in an array of techniques employed in a multi-modal imaging approach to cultural heritage studies [107].

## 3. Edge-illumination in the context of other x-ray phase contrast imaging techniques implemented with laboratory sources

The key limitation of EI is the flux reduction caused by the absorbing nature of the used masks. If masks with a 50% open fraction are used, a relative misalignment corresponding to the 50% of the IC (approximately the position of maximum phase sensitivity) leads to rejecting approximately 75% of the flux. This applies to single- and double-shot acquisitions, while three-shot acquisition aimed at retrieving also the dark field channel improve things slightly as the third image is acquired with the masks aligned (top of the IC), reducing the flux rejection to 66%. In truth, masks with smaller open fractions are often used to maximise sensitivity and increase the spatial resolution (or, in the case of ‘skipped masks’, to mitigate the negative effects of pixel cross-talk), leading to proportionally higher flux reductions. It is important to note that this applies to flux but not to the dose delivered to the sample, since the latter is largely protected by the pre-sample mask. While the ‘standard’ 50% misalignment leads to 50% dose efficiency, it has been repeatedly proven that the same image quality can be obtained by proportionally reducing both the aperture size in the pre-sample masks and relative misalignment between the masks, leading to a better dose efficiency for the same flux efficiency [62].

These limitations in terms of flux reduction are accompanied by a series of advantages, some of which are unique to EI. First and foremost, EI significantly relaxes the requirements on the source’s spatial coherence, as it allows the use of uncollimated and unapertured focal spots of at least 100 *μ*m [108] while maintaining a phase sensitivity comparable to gratings-based methods [54]. Their relatively large feature size makes mask fabrication simpler, to the extent that lithographic processes are not necessary, and substrate-free masks can be fabricated through laser ablation in e.g. tungsten foils [86]. The same property simplifies mask alignment, and makes it easy to have approximately the same illumination level (i.e. same position on the IC) for all pixels over the entire field of view, with any remaining small differences easily corrected by flat fielding. Combined with single-shot, Paganin-style phase retrieval [17], this allows keeping the masks at a fixed position during CT acquisitions, i.e. it allows flyscans, which led to some of the fastest (3’) phase CT acquisitions performed with conventional sources [80]. EI can be implemented with detectors with any pixel size and PSF, while allowing flexible, user-defined resolution de-coupled from focal spot and detector characteristics, as this is determined by the apertures in the pre-sample mask [57]. While in principle reaching aperture-driven resolution requires dithering, which lengthens acquisitions and is not compatible with flyscans, the recently developed cycloidal CT method, in which the sample is roto-translated in a structured beam, offers a possible solution [109]. Finally, the use of a pre-sample absorbing mask makes it easy to keep the delivered dose under control [62, 68]. The increased awareness of these advantages is leading to EI being adopted by labs worldwide e.g. [110–115]; to the best of our knowledge, there are currently five more labs in Europe currently installing EI systems, some of which have already explicitly declared this intention [116].

In terms of flux efficiency, Talbot-Lau methods also feature two absorption gratings, and are therefore subject to similar flux losses. While transition to graphite substrates has been considered [117], the absorption in silicon substrates should also be taken into account since, at least for some applications, it can be considerable. A study that looked at proposed setups for mammography [114] concluded that 10% and 1.4% of the photons emitted by the source are detected by EI and gratings setups, respectively; a modified EI setup with reduced open fraction for dose minimization was considered in that case. Considering that two gratings are normally placed downstream of the sample, this has consequences also on dose delivery.

An improvement in terms of flux efficiency comes with the use of a single (pre-sample) mask. In this case, the flux efficiency corresponds to the mask open fraction, and the method becomes 100% dose efficient. However, it requires the use of specific detectors, which means sacrificing the ability to use any detector technology. A first approach consists in the use of a detector with a sharp PSF, like that provided by some direct conversion detectors, as the ‘refraction sensing’ mechanism [118–120]. This approach has been used since some of the earliest embodiments of EI with synchrotron radiation (e.g. [52]). In a laboratory embodiment, it consists of aligning the beamlets created by a skipped sample mask so that they straddle neighbouring pixels. The method works well with photon counting detectors, especially if mechanisms to handle charge sharing effects are available (e.g. [121]). These detectors, however, are currently only available in small areas, mostly for scientific use. With more widely available, larger area direct conversion solutions such as those based on amorphous selenium, the reduced steepness of the PSF slopes rapidly leads to a reduction in refraction sensitivity [122].

A simpler alternative consists in using a detector with a sufficiently small pixel to resolve the beamlets created by the sample mask [123], effectively an x-ray embodiment of the Hartmann wavefront sensor [124]. This approach was shown to be easily adaptable to laboratory sources [125], including with limited coherence [126]. This Hartmann-like ‘beam tracking’ approach has the great advantage that, since beamlets are individually resolved by the detector pixels, attenuation, refraction and dark-field can be all retrieved from a single frame; moreover, no mask alignment is necessary. A significant downside, however, is the need for a detector with a small pixel size: this poses significant limits on the field of view, since detectors typically feature 2k pixels per side, as well as on detection efficiency, since the realisation of small pixel detectors with high quantum efficiency is a significant technological challenge [127]. This translates directly into inefficiencies in both flux and dose delivery. The same limitations are shared by methods where a mesh instead of a mask is placed on the beam path [128].

In terms of flux efficiency of the used optical elements, the best results are provided by ‘speckle’ methods, where the reference ‘speckled’ pattern created by a phase modulator with negligible absorption, such as a sheet of sandpaper [129], is tracked before and after the introduction of a sample. Provided a thin enough substrate and/or x-rays of sufficient energy are used, similar results can be obtained with a single phase grating [130]. Alongside requiring a small pixel size to resolve the speckles, which as discussed imposes limitations on field-of-view and detection (and therefore dose and flux) efficiency, this method also requires spatial coherence for the speckles to become visible. Outside synchrotrons, this has so far imposed the use of micro-focal [131] or ‘liquid metal jet’ x-ray sources [132, 133]. Effectively, these limitations are comparable to those of PB XPCI, and indeed excellent PB images were obtained using liquid metal jet sources [134]. An additional concern is the need to preserve the modulation when traversing samples which are not thin phase objects (which became an issue also with phase gratings when the approach was scaled up to image porcine lungs [135] and subsequently humans cadavers [136], requiring a switch to absorption-only gratings). Finally, scattering samples often create speckles themselves, which may become indistinguishable from those created by the modulator. This notwithstanding, the method has potential in specific areas such as the histological analysis of soft tissues.

Some of the above issues could be circumvented by creating a ‘speckled’ reference image by using an absorbing structure [137, 138], effectively the equivalent of an ‘irregular’ sample mask, instead of a low-absorbing phase modulator. Although this may face some of the limitations mentioned below in relation to non-optimised positioning of the absorbing elements, progress in this area can be facilitated by current effort dedicated to the development of advanced retrieval algorithms (e.g. [139–141]).

Finally, we mention a ‘hybrid’ between EI and grating interferometry proposed by Huang *et al* [142], in which a grating-style setup completely based on absorption gratings was developed. Because the pitch of the grating was not matched to the detector pixels, a grating-style retrieval had to be adopted, which entailed scanning the gratings over an entire period rather than positioning them at the locations resulting in the highest phase sensitivity, as done in EI. This resulted in a reduced sensitivity [143] compared to both the gratings and EI methods. A detailed analysis of how the masks’ positions affect sensitivity in EI, and consequent recipes for optimal positioning, are provided in [144].

## Data Availability

No new data were created or analysed in this study.
